# Application of Polymer Membranes for a Purification of Fuel Oxygenated Additive. Methanol/Methyl Tert-butyl Ether (MTBE) Separation via Pervaporation: A Comprehensive Review

**DOI:** 10.3390/polym12102218

**Published:** 2020-09-27

**Authors:** Alexandra Pulyalina, Valeriia Rostovtseva, Ilya Faykov, Alexander Toikka

**Affiliations:** Institute of Chemistry, Saint Petersburg State University, Universitetskiy pr. 26, 198504 Saint Petersburg, Russia; v.rostovtseva@spbu.ru (V.R.); st022544@student.spbu.ru (I.F.); a.toikka@spbu.ru (A.T.)

**Keywords:** polymer membrane, mixed matrix membranes, pervaporation, MTBE, azeotropic mixture, fuel additive

## Abstract

Methyl Tert-butyl Ether (MTBE) remains the most popular fuel additive to improve fuel performance and reduce the emission of hazardous components. The most common method of MTBE production is a catalytic synthesis with a great excess of methanol to improve the reaction yield. The problems of obtaining pure MTBE from the final product have determined the search for new techniques; primarily membrane methods. Pervaporation as an optimal membrane process for highly selective separation of organic mixtures is of particular interest. This review is focused on analysis of the research works on the various polymer membranes and their efficiency for the separation of the azeotropic methanol/MTBE mixture. Currently the most popular materials with optimal transport properties are poly(vinyl alcohol), cellulose acetate and polyheteroarylenes. Mixed matrix membranes (MMM) are highly effective as well as they show overall operational stability.

## 1. Introduction

The growing interest in the development of highly environmentally friendly transport fuels, and in particular, motor fuel, has led to a number of problems associated with the development of resource and energy-saving technologies used in their production. Despite the search and the proposed options for alternative fuels, the focus in coming years will be on fuel based on hydrocarbons, i.e., gasolines. It is well known that one of the most important negative factors in the use of gasoline, along with the inevitable combustion products (carbon dioxide and carbon monoxide), is environmental pollution with octane additives that improve fuel performance, but which are dangerous in terms of their impact on human health. A significant step in this regard was associated with the replacement of cheap lead additives (tetraethyl lead and its analogues) with safer compounds. It should be noted that, at present, the use of gasolines containing tetraethyl lead is prohibited in a number of countries [1]. Ethers are currently used as some of the most effective additives for the production of more environmentally friendly high-octane fuels. These include methyl tert-butyl ether (MTBE), ethyl tert-butyl ether (ETBE), tert-amyl methyl ether (TAME) and others. Other compounds such as ethanol and isooctane are also used for this purpose. Among the alternatives of non-lead additives, MTBE remains the most popular and usable one in the industry despite the well-known discussions related to its low biodegradability (in comparison with ETBE) and other negative characteristics (see, e.g., [2]).

Due to its chemical composition and high oxygen content, MTBE greatly affects detonation properties and makes it possible to reduce gasoline consumption in combination with a modern fuel supply system of combustion chamber. The most common method of MTBE producing is a catalytic synthesis, which implies the use of isobutylene and methanol as the main precursors. In order to obtain the maximum yield of the reaction product, an excess of methanol is often used. A large amount of alcohol (about 20 wt.%) is therefore present in the final product, the release of which into the atmosphere harms both environment and human health. Table 1 lists some physical properties of the studied liquids.

**Figure 1:**
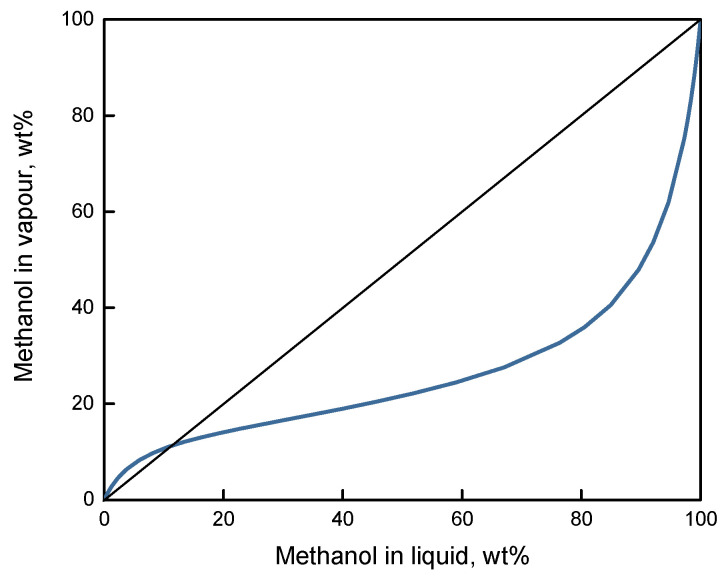
Vapor–liquid equilibrium curve for the MTBE-methanol system at 293.15 K [11].

Traditional methods of this mixture separation are primarily associated with the use of reactive and azeotropic distillation [4,5,6]. One of the known problems is caused by an azeotrope formation in the MTBE-methanol binary system: according to various authors, it contains from 80 to 70 mol. % MTBE in the temperature and pressure range of 298.15–324.3 K and 36.24–100.50 kPa, respectively [7,8,9]. The vapor–liquid equilibrium diagram for 293.15 K is presented in Figure 1. It shows equilibrium vapor–liquid compositions of the methanol-MTBE system at the given temperature and variable pressure. The intersection point of vapor–liquid equilibrium curve and diagonal line corresponds to the azeotropic point of this system at given conditions. Modern methods of distillation make it possible to solve the problem of separating complex mixtures. However, in some cases, their application is associated with additional problems of energy- and resource-saving [10]. In particular, when using azeotropic distillation, additional tasks arise of purifying the product from the separating agent, and other technological challenges also occur.

In the industry, methanol impurities have been recently removed by mixing the final product with water and then extracting MTBE. The remaining aqueous methanol solution is subsequently distilled to recover and recycle alcohol [12]. However, this process is expensive and energy-consuming, and also leads to the loss of target components.

**Figure 2:**
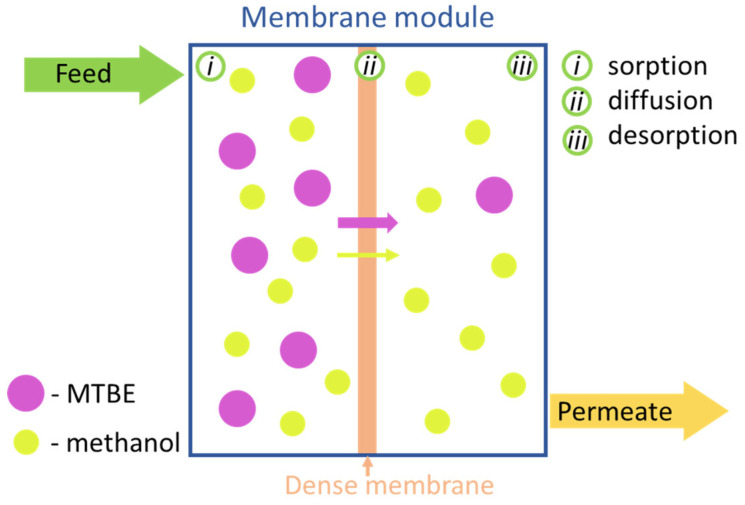
Pervaporation mechanism.

The problems of obtaining pure MTBE from the final product have determined the search for new techniques, primarily membrane methods. Pervaporation as an optimal membrane process for highly selective separation of organic mixtures is of particular interest. The scheme of the pervaporation separation mechanism is shown in Figure 2. The solution–diffusion model is widely used to describe mass transfer in the case of dense polymer membranes for pervaporation. According to this mechanism, the pervaporation process includes three successive stages: solution/sorption of feed molecules at the upstream side of the membrane; diffusion of permeate through the membrane; and desorption/evaporation of permeate into the vapor phase at the downstream surface of the membrane. The differences in the sorption degree of permeating molecules, as well as in their diffusion rates, provide permselective separation. The mass transport in this case can be described using solubility and diffusivity parameters. The first one is a thermodynamic parameter which means the amount of component sorbed on the membrane surface under equilibrium conditions. It is primarily determined by the interaction between permeate molecules and the membrane matrix through the possible formation of hydrogen bonds, dipole–dipole interactions or by dispersion forces. The second one is a kinetic parameter depending on the size and shape of permeating molecules and features in membranes structure. Thus, the investigation of morphology, physical parameters of the membranes (density, crystallinity, surface characterization, solubility parameters etc.) as well as the study of sorption and diffusion parameters are necessary to explain the transport properties of the membranes.

Scientific and technological tasks of pervaporation separation of organic mixtures are primarily focused on the development of effective organoselective membranes for removing methanol from its mixture with MTBE [13].

This review provides a brief analysis of the research works devoted to the study of various polymer membranes and their efficiency for the separation of methanol/MTBE azeotropic mixture. As a rule, these membranes are based on hydrophilic polymers such as cellulose acetate (CA), cellulose triacetate (CTA) [14,15,16,17,18,19,20,21], polyvinyl alcohol (PVA) [22,23,24,25] and others. Recent researches are also aimed at solving the problems of improving the mechanical strength, overall operational stability of membranes, and traditional pervaporation tasks of increasing the separation efficiency while maintaining a sufficiently high transmembrane transport rate, that is, the technological performance of membranes. As far as it is known there is a commercial membrane designed for pervaporation removal of methanol from volatile organic mixtures, namely, PERVAP™ 4155 (formerly PERVAP^TM^ 2255) composed of a PVA-selective layer supported by a polyacryl nitrile (PAN) layer and a non-woven material. Such membranes with different cross-linking degrees of PVA chains were successfully studied for methanol/methyl acetate [26], methanol/trimethyl borate [27], the transesterification reaction between methyl acetate and butanol to produce methanol and butyl acetate [28].

In the course of the discussion, the separation factor, which is an important membrane transport parameter and is a measure of the efficiency of the separation process, will be used constantly to compare membrane performance. The separation factor β is calculated as follows:

(1) $$\beta =\frac{\left[y/\left(1-y\right)\right]}{\left[x/\left(1-x\right)\right]}$$

where *x* and *y* are the methanol concentrations in the feed and the permeate, respectively.

## 2. Polymer Membranes for Methanol/MTBE Separation

### 2.1. Cellulose Acetate

Cellulose acetate (CA) is one of the most popular membrane materials. This natural polymer has good film-forming properties and ease of processing, it is a harmless and renewable material, which makes it especially exploited in many technologies [29], including pervaporation separation of a methanol/MTBE mixture. However, the performance of a membrane based on pure CA is low; therefore, modern research is aimed at modifying this material to increase the efficiency of the process.

In one of the earliest works [14], CA and cellulose triacetate (CTA) membranes were used for experiments on pervaporation of methanol/MTBE mixture. It was found that a CA-based membrane had a relatively low total flux in comparison with a CTA membrane, but its selectivity was of a higher value. According to the results of sorption experiments, it was noted that sorption degree practically did not change with an increase in the volume fraction of methanol in the feed. This fact was interpreted by the authors as an unusual phenomenon, since methanol forms hydrogen bonds with the acetates under the study. In order to explain this behavior, the authors used the relations of the Flory–Huggins theory (for component activities) and the solution–diffusion model. The activity values were determined by the equations of the UNIFAC group model. It was found that the volume fraction of methanol in membrane increases with its volume fraction in the feed whereas that of MTBE decreases with increasing volume fraction of methanol in the feed for both CA and CTA. These two opposite dependences make the swelling ratio independent of the volume fraction of methanol. The results of calculations of the Flory–Huggins interaction parameters including their dependence on concentration, in the view of the authors, give a definite explanation of the indicated feature of pervaporation results, and are also consistent with experimental data on the swelling of CA and CTA in methanol/MTBE solutions.

Tabe-Mohammadi et al. [15] studied the characteristics of cellulose acetate membranes prepared using acetone, dimethylformamide (DMF) and N-methylpyrrolidone (NMP) as solvents. The authors determined the dependence of the transport properties of the membranes on the solvent nature. When using DMF as a solvent, the membrane showed the highest selectivity and the lowest normalized flux. The differences in membrane performance were explained by the influence of solvent evaporation rate. The authors suggested that there is an optimal solvent boiling point at which the evaporation rate is low enough to form a stable and dense membrane, and high enough for complete removal of the solvent. The relationship between the polymer concentration in the solution and morphology of the resulting membranes was also studied. With an increase in polymer concentration and almost identical film-forming conditions, the membrane structure changed from a porous to a dense one. At the same time, an increase in selectivity and a decrease in membrane permeability were observed with an increase in polymer concentration in the solution.

Polymer membranes from CA and poly (N-vinyl-2-pyrrolidone) (PVP) blends of various compositions were studied by Wu et al. [16]. As is known, techniques based on the use of polymer blends often allow one to change significantly the transport properties of membrane materials, in particular, increasing permeability while maintaining selectivity, and also improving such operational characteristics as mechanical strength, thermal stability and physical properties. In this work, the study of mechanical stability showed that the addition of more than 10 wt.% PVP to the CA leads to an improvement in the mechanical properties of the membranes. The analysis of the contribution of sorption and diffusion components in selectivity showed that the preferred transport of molecules was determined primarily by diffusion rather than sorption. It was also shown that the total flux as well as the separation factor increased with the addition of PVP, when the PVP content in the blend was 10–15 wt.%. The membrane containing 85 wt.% CA and 15 wt.% PVP had the maximum separation factor (411) and total flux of 430 g m^−2^ h^−1^ for the feed mixture containing 20 wt.% methanol at 313 K.

In the work [30], Cao et al. studied the effect of acetylation degree of cellulose acetate on pervaporation characteristics. Such modification of the material was aimed at creating more hydrophilic membrane and improving its transport parameters. The authors led to the negative conclusion regarding the low permselectivity of the membranes based on pure cellulose acetate in separating methanol from its mixture with MTBE.

Niang and Luo carried out a somewhat controversial, from our point of view, analysis of pervaporation including estimation of the interaction of methanol and MTBE in the azeotropic mixture [31]. The conclusions were made, among others, based on sorption and pervaporation experiments using CTA membranes. Sorption experiments showed that with an increase in methanol feed concentration, the membrane swelling degree gradually increased until a certain maximum value was reached. The authors suggested that methanol selective sorption is associated with the formation of hydrogen bonds between the carbonyl groups of CTA and the hydroxyl group of methanol. It was noted that the interaction between penetrant molecules plays an important role in the separation process, especially in the case of azeotropic mixture. At the azeotropic point, the interaction between the methanol and MTBE molecules reaches a maximum which leads to a significant decrease in methanol sorption. At other concentrations, these interactions become weaker and methanol mainly interacts with the polymer membrane. As a result, methanol replaces MTBE molecules between the polymer chains, which leads to an increase in selectivity with respect to methanol. From our point of view, the statement about the maximum interaction between components in an azeotropic mixture is not correct. The formation of azeotrope and its composition are not only related to the degree of intermolecular interaction, but are also determined by other factors, such as the ratio of boiling points (vapor pressure) of pure substances. In addition, positive deviations from ideality (respectively, a positive azeotrope, with a minimum boiling point) indicate a weaker interaction of the substances of the mixture with each other compared to the intermolecular interaction in the pure components.

Some additional aspects related to the separation features of the methanol/MTBE system were considered in paper [21]. Pervaporation was carried out at various compositions and temperatures of the feed mixture. The aim of the work was to study the patterns of permeability behavior of the dense CTA-based membranes. The obtained experimental data indicate that the effect of plasticization influenced the performance, selectivity and activation energy of the process. The plasticization effect results in coupling transport of the penetrants: the flux of one component of a binary mixture is influenced by the other component, which causes membrane matrix relaxation. The results showed that the components of mixtures of various compositions diffused through the membrane at different rates depending on plasticization and temperature. In general, it was noted that plasticization has a great effect on the ability of membranes to swell and sorb. Thus, it was found that with the increasing methanol content in the feed, the swelling degree raises until it reaches a maximum due to a strong plasticization effect of methanol and MTBE molecules. This, in turn, leads to a change in membrane transport properties.

Niang et al. [20] devoted their research to the features of pervaporation membranes based on the blends of CA and cellulose acetate hydrogen phthalate (CAHP) of different compositions. The optimal composition of the membrane material was determined by studying the series of samples with cellulose acetate content from 10 to 65 wt.% in separating the mixture containing 20 wt.% methanol. The membrane containing 30 wt.% CA had the best selective and transport characteristics: the selectivity was about 100, while the total flux was 2000 g/m^2^h (for the mixture containing 20 wt.% methanol). The effect of temperature on the operational parameters of the membranes was studied in the range from 30 to 50 °C for various feed concentrations (from 5 to 30 wt.% methanol). A decrease in selectivity and an increase in total flux with increasing temperature were noted. The temperature effect is most significant at low methanol content in the feed. One of the main results of the work, from the point of view of the authors, [20] is as follows: the possibility of increasing the efficiency of pervaporation separation when using polymer blends and varying their composition is shown. Membranes based on cellulose acetate phthalate was also used in [20]. They have good separation ability; however, there are certain difficulties in obtaining hydrophthalate from cellulose acetate, which significantly limits the application of such materials.

Table 2 shows the main transport characteristics of the membranes based on CA in pervaporation of methanol/MTBE mixture. It can be seen that the membranes based on CA blends with other polymers have the best separation performance in this case.

### 2.2. Polyvinyl Alcohol (PVA)

Membranes based on polyvinyl alcohol (PVA) are also widely used in membrane technology including the separation of methanol/MTBE mixtures. This hydrophilic polymer is most often used for the dehydration of organic mixtures. For pervaporation application of PVA, it is usually crosslinked to reduce membrane swelling and increase operating time. Swelling, as a rule, leads to an increase in permeability, however, it can significantly reduce the selectivity; in addition, it may lead to formation of the defects as well as peeling of PVA from the substrate in the form of a thin selective layer. Crosslinking of PVA can be performed by incorporating a functional component or by thermal and chemical treatment.

The works [22,32,33] were devoted to the membranes based on the blends of two polymers: PVA and poly (acrylic acid) (PAA) containing from 10 to 30% PVA. The ability to control the transport properties of the membranes by changing the polymer blend composition (10 to 30% PVA) was shown in [22]. As the PVA content in the blend increased, the total flux gradually decreased, and the selectivity increased for all compositions of the feed (5–20 wt.% methanol). When comparing the individual fluxes of the components through the membrane, it was noted that the transport of methanol molecules is predominant. In [32], membranes with a functional group ratio (–COOH/–OH) within the range of 0.5–2.5 were preferably permeable towards methanol. The maximum total flux and selectivity was observed at a ratio of –COOH/–OH about 2, and subsequently these parameters decreased. The highest values of the separation factor and total flux were about 300 and 0.13 kg/m^2^h, respectively. The authors of paper [33] also carried out sorption and pervaporation experiments for the cross-linked PVA/PAA membranes. As the PAA content in the membranes increased, the solubility of the components and fluxes decreased while the selectivity increased. The authors attribute this to the reduced mobility of the polymer segment as a result of cross-linking, which leads to reduced diffusion of penetrants through the membrane. The polar nature of the methanol molecule determined its predominant transport through the PVA/PAA membrane which is consistent with the works discussed above. As the amount of cross-linking agent increased in the membrane, a decrease in the swelling degree was observed.

Rhim et al. studied the transport characteristics of a PVA-based membrane using poly (acrylic acid) (PAA) and sulfosuccinic acid (SSA) as crosslinking agents of PVA [23]. The effect of operating temperature and the amount of crosslinking agent on the membrane separation properties was investigated. The authors concluded that the total flux through the membrane is affected by structural changes during crosslinking as well as by the formation of hydrogen bonds between carboxyl groups, PVA molecules and the components of the feed solution. It was found that the sulfuric acid group in SSA took an important role in the membrane performance. The authors noted that the effect of the structural change, i.e., more compact network due to the cross-linking reaction, increased with more addition of SSA, but the hydrogen bonding effect with the solvents was reduced. The cross-linking effect might be over the hydrogen bonding effect due to the sulfuric acid group in 3 and 5% SSA membranes, and these two factors act vice versa in 7% SSA membrane. The highest separation factor (2095) in the case of mixture containing 20 wt.% methanol (at 30 °C) was obtained for the membrane containing 5% SSA, with the total flux value of 12.79 g/m^2^h. However, the authors provided no information on the swelling and transport properties of pristine PVA membranes; thus, there is no possibility to estimate the significance of structural changes due to cross-linking effect.

The membranes based on a PVA/CA blend with a CA content ranging from 0 to 100 wt.% were studied in [34]. As the CA concentration increased, the fractional free volume of the membrane, the hydrophilicity, and the amorphous phase content in the membrane structure also increased. In addition, it was noted that under certain conditions and concentrations two-phase regions are detected in the membrane structure, which are clearly visible, in particular, in SEM micrographs. The detailed analysis of such phase separation is also presented in the paper [31]. The resulting membranes had good separation performance in the pervaporation of methanol/MTBE mixture when the ratio of the polymer components was of 15:85 wt.%. It was shown that the use of the polymer blends improved membrane permeability (compared to pure PVP) and its selectivity (compared to pure CA).

Peivasti et al. [35] estimated the influence of process conditions on the transport parameters of PVA membranes. The experimental results were obtained at various concentrations of methanol in the feed (10–30 wt.%), temperatures (25–45 °C) and pressures (15–35 mbar). It was found that the separation factor of the PVA membrane for methanol is higher than for MTBE. It was also noted that an increase in the feed concentration of methanol enhances membrane swelling. Both the total flux and separation factor increased with decreasing residual pressure under the membrane, which had a positive effect on process efficiency. As a result, it was concluded that a relatively high vacuum and low temperature are preferable for the separation of this organic mixture.

Composite membranes based on PVA as a selective layer and polyacrylonitrile (PAN) or CA as a substrate material were prepared in the work [24]. When separating a mixture containing 7 wt.% MTBE, the efficiency of the composite membrane with CA (with MWCO 0.5 × 10^4^) as a substrate is higher than that of the membrane with PAN (with MWCO 5 × 10^4^). The authors attribute these results to the more uniform structure of the selective PVA layer on the CA substrate with a smoother and more even surface. The total fluxes through the PVA/PAN and PVA/CA composite membranes were more than 487 and 803 g/m^2^h, and the methanol concentration in the permeate reached more than 99.3 and 99.5 wt.%, respectively.

Singha and co-authors [36] studied PVA that was chemically modified by crosslinking it with a) a copolymer of acrylic acid (AA) and hydroxyethyl methacrylate (HEMA) in an aqueous solution of PVA and b) glutaraldehyde to obtain a membrane with an interpenetrating polymer network (IPN) with the different PVA: Poly(AA-co-HEMA) (PolyAH) ratios (1:0.25, 1:0.50 and 1:0.75) [36]. It was shown that the permeability and selectivity of methanol for all obtained membranes increased with increasing the amount of copolymer in the PVA matrix. At the same time, the membrane containing 50% PolyAH (PVAHII) showed the optimal characteristics both in terms of the permeability and selectivity. Meanwhile, the membrane containing 75% PolyAH (PVAHIII) showed the highest flux, but the selectivity towards methanol was lower compared to PVAHII, which was associated with an increase in the fractional free volume in the membrane structure. The authors compared the obtained membranes (PVAHII and PVAHIII) with other membranes used for the separation of methanol/MTBE mixtures: it is indicated that the developed membranes have significantly improved selectivity and fluxes values compared to other membranes (Table 2 of the paper [36]). However, the same table shows, for example, the data for the membrane based on polyphenylene oxide (PPO-OH) [37], which has the best indicated characteristics.

It should be noted that the preparation of polymer blends based on PVP leads to obtaining the most optimal transport properties (Table 3). Thus, the greatest separation factor is achieved for PVA/CA and PVA/PAN composite membranes, while the PVA/CA bland has the highest permeability.

### 2.3. Chitosan

Chitosan (beta-(1,4)-2-amino-2-deoxy-d-glucose) (CS) is a deacylated form of chitin, a compound that is mainly obtained from the cuticle of crustaceans. It contains both hydroxyl and amino groups, which makes it easily modifiable and attractive for use as a membrane material [38]. It has been proven that CS has good film-forming properties, chemical resistance and high permeability [39]. However, poor physical and mechanical properties and high sorption degrees of polar liquids limit its practical application. One way of effectively overcoming these limitations is chemical crosslinking. It gives opportunities for the usage of membranes based on chitosan for the separation of methanol/MTBE mixtures.

Polyion complex (PIC) composite membranes consisting of sodium alginate (SA) and chitosan were obtained by alternating the electrostatic adsorption of SA and chitosan on a polysulfone substrate in the works [40,41]. For the membranes obtained from the polymer solutions of different concentrations, morphological differences were especially noticeable in SEM micrographs. In addition, the amorphous nature of the PIC membranes, elongation at break and elastic modulus varied significantly depending on the concentration of the polymer solution. The pervaporation results showed that the membranes were selective towards methanol. In addition, with an increase in the chitosan content, the penetration rates and the MTBE permeate content gradually decreased due to an increase in the complexation degree between SA and chitosan and the formation of a compact network structure.

Cao et al. [42] obtained and investigated the membranes from a mixture of CS with PVP. The data of IR spectra for hydrogen interactions of carbonyl and hydroxyl groups indicated good compatibility of the polymers. The preferred diffusion of methanol was typical for the entire series of the obtained membranes, while the MTBE flux increased slightly with increasing the PVP content in the membrane. Because PVP forms hydrogen bonds with the hydroxyl group of the chitosan chain, the rearrangement of the polymer chains occured resulting in a decrease in the interaction between the chitosan chains and the appearance of a greater number of accessible chitosan hydroxyl groups interacting with MTBE. These considerations fit in well with experimental data on pervaporation: with an increase in the PVP content (0.13–0.5%), the total flux through the membrane increases, and the separation factor decreases.

The influence of different anionic surfactants on pervaporation properties of CS-based membranes was also investigated in [43]. It was established that pervaporation characteristics of surfactant modified CS membrane were substantially improved due to the decreased membrane thickness and possible enhanced affinity towards methanol. Thus, the thickness of the complex chitosan layer was about 2 μm when adding dioctyl sodium sulfosuccinate (DSS), whereas that of pure (CS) layer was about 10 μm. Moreover, upon the addition of surfactants, it was found that there were conformational changes of polymer chains. Among the used surfactants (sodium dodecyl sulfate (SDS), sodium laurate (SL), sodium stearate (SS), DSS and amphoteric sodium N-lauroyl sarcosinate (SLS)), CS-DSS membrane showed the highest flux for 20% MeOH/80% MTBE mixture at 25 °C, and the methanol content in the permeate was about 95%.

The efficiency of chitosan composite membrane modified by sulfuric acid and four surfactants at different methanol contents in the feed, temperatures and cross-linking degrees was studied in [44]. For the membrane containing surfactants the methanol concentration in the permeate was 98.3 wt.%, and the total flux reached 470 g/m^2^h at 25 °C. With the increasing of temperature, these parameters changed to 97.8 wt.% and 1170 g/m^2^h, respectively. The authors attempted to explain this effect by the fact that the ionic nature of the composite sodium lauryl sulfate (SLS) membrane enhanced the penetration of polar methanol molecules through the membrane. Moreover, linear alkyl chains of the surfactants can increase the free volume fraction in the chitosan membrane and prevent the penetration of relatively bulky MTBE molecules. Thus, the chitosan-SLS membranes have higher permeability with the high separation factor compared to other chitosan-surfactant membranes (sodium lauryl ether sulfate (SLES), sodium trideceth-7 carboxylate (STC), disodium cocamido mipa-sulfosulccinate (DCMSC)). It is worth noting that chitosan-SLS membrane has the best performance among those presented in this section (Table 4).

### 2.4. Polyarylethersulfone (PES)

Polyarylethersulfone with cardo (PES-C) is a novel polymer constructional material which is poly(ethersulfone) containing a rather bulky and polarizable phenolphthalein group substituting the oxygen atom [45]. It is a heat-resistant polymer characterized by high mechanical properties and chemical resistance. The presence of the cardo-group in the PES-C backbone reduces the crystallinity of the polymer, therefore making it more soluble in polar organic solvents which are currently used for membrane manufacturing. Due to the excellent properties mentioned above, PES-C and its derivatives were used as membrane materials, in particular for pervaporation of the methanol/MTBE mixture.

Blanco et al. [45] devoted their research to the membranes based on the blend of PES-C and PVP. When studying sorption and diffusion selectivity, it was shown that the transport of separated components through pervaporation membranes is limited mainly by diffusion. With an increase in the PVP content to 16 wt.%, the total flux and the separation factor of the membrane also increased, reaching 889 and 3.44 kg μm/m^2^h, respectively (at 40 °C).

The membranes based on PES-C with high chemical resistance and moderate mechanical properties were obtained and studied in the work [46]. NMP was used as a solvent, the use of which was preferable in comparison with DMFA. The swelling degree of the membrane in methanol/MTBE mixtures was less than 7 wt.%, which is associated with the high resistance of PES-C to these solvents. The results of studying the impact of temperature and annealing time showed that their increase leads to a compactification of the membrane structure and, as a result, an increase in the selectivity for methanol and the decrease of permeability. With an increase in the methanol content in the feed solution from 5 to 40 wt.%, the total flux increased from 1.21 to 4.52 kg/m^2^h. The permeate contained almost pure methanol (above 98.5 wt.%).

Based on the data presented in this section, we can conclude that the developed membranes from sulfonated polyarylethersulfone with cardo [47] allow the highest separation selectivity (Table 5) to be achieved. Blending PES-C and PVP contributes, in turn, to the highest permeability.

### 2.5. Polyheteroarylenes

Polyheteroarylenes are a group of very strong, thermo- and chemically-resistant polymers. They possess high operational stability including optimal mechanical properties, resistance to solvents and other characteristics which determine their promising practical applications. The transport, selective and physico-chemical properties of membranes based on polyheteroarylenes have been extensively studied, but only a few papers have been published on their use in the separation of methanol/MTBE mixtures over the past decade [48].

Polyamide-6 (PA-6) was used to prepare homogeneous, composite and hybrid membranes containing zirconium oxide [49]. The authors of [49] note, according to AFM images, that the hybrid membranes have a higher roughness compared to the polymer membrane. Based on the TEM analysis, it was confirmed that the metal oxide particles are uniformly distributed in the polymer matrix. Furthermore, the results of XRD analysis showed that PA-6 exists in the crystalline phase. In the case of separation of methanol/MTBE mixture at the concentration ratio of 50/50, the separation factor for the PA-6-based membrane modified by 10 wt.% ZrO_2_ had the highest value, at 48. The normalized methanol flux for this membrane was 12.4 kg/m^2^h. On the basis of the AFM results it can be concluded that with an increase in membrane surface roughness, membrane separation factor decreases (unmodified PA-6 has the highest separation factor value and the lowest average roughness). This may result from higher sorption activity of MMMs towards both methanol and MTBE. It is also indicated that the presence of water as an impurity in the feed causes a significant decrease in the methanol transfer rate through the PA-6 membrane.

In the study [50], Castro-Muñoz et al. successfully tested the membranes based on another polyheteroarylene, Matrimid^®^, in pervaporation separation of the azeotropic methanol/MTBE mixture. The SEM images showed that the membranes have a uniform and smooth surface with no signs of plastic deformation. When studying the mechanical characteristics of the membranes, it was found that after the membrane immersion in the methanol/MTBE solution, the strength parameters remained practically unchanged which indicates the stability of this material in relation to the components of the separated mixture. The most optimal transport properties for membranes on the base of Matrimid^®^ were obtained at 45 °C and a residual pressure of 0.054 mbar: the total flux and the separation factor were about 0.073 kg/m^2^h and 21.16, respectively.

The investigation of Matrimid as a membrane material was continued in the work [51]. The authors developed hybrid membranes with the addition of graphene oxide (GO) up to 4 wt.% and studied their mechanical and transport properties. It was noted that the maximum total flux (0.091 kg m^−2^ h^−1^) was obtained for the membrane containing 4 wt.% GO, but the membrane with 1 wt.% GO had the best separation factor. However, when comparing these results with the published data on other similar membranes, the developed hybrid membranes are less effective. Nevertheless, it should be noted that rather low amounts of modifier (1–4 wt.%) were introduced in the membranes in this work, while the modifier content is from 10 to 30 wt.% in most studies. In general, performance of MMMs depends on the filler nature, its content in a membrane, as well as the properties of the polymer matrix. For example, with the increasing of GO loading in this work, an increase in the total permeation rate was observed. This may be a result of the free volume increase as well as possible interfacial selective gaps between GO flakes and polyimide matrix, while the highly hydrophilic nature of the filler can also produce an increase in the permeation rates by preferential adsorption of the more polar compound (e.g., methanol). In this case, it would be useful to investigate MMMs with higher GO content and compare their performance with that of similar MMMs also based on Matrimid.

Alibakhshian et al. [52] obtained polyamide by interfacial reaction of *m-*phenylenediamine with trimesoyl chloride and used it as a thin selective layer for composite membranes with different types of substrates: polyethersulfone, polyetherimide, cellulose acetate and polyacrylonitrile. The authors assumed that the selection of a suitable substrate is able to provide high performance. Based on the analysis of the pure water flux values, mechanical properties and membrane morphology, the authors of [52] noted that the membrane with the polyethersulfone substrate has the most optimal properties. This membrane has the total flux of 0.453 kg m^−2^ h^−1^ and the separation factor of 73 in the separation of the methanol/MTBE mixture (20/80 wt.%, 30 °C). Compared with the other data published on this subject, the selectivity values remained rather high at high fluxes for the membranes developed in this work (see Table 6).

**Figure 3:**
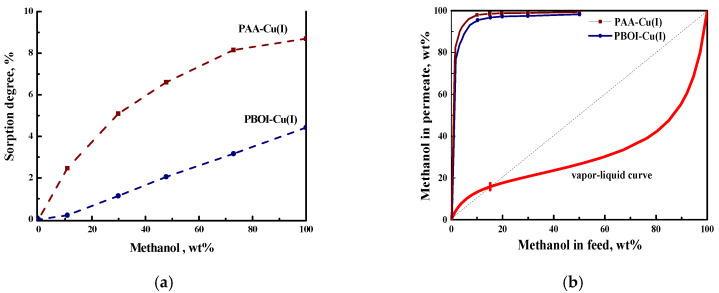
Dependence of (**a**) equilibrium sorption degree and (**b**) methanol concentration in permeate on methanol concentration in feed methanol/MTBE mixtures for PAA-Cu(I) and PBOI-Cu(I) membranes, 20 °C [53].

A comparative study of the metal-polymer complexes based on polybenzoxazinoneimide (PBOI) as well as its hydrolytically stable prepolymer-imide-containing polyamic acid (PAA) and monovalent copper as new membrane materials were described in paper [53]. Both polymers have high thermal stability and mechanical strength. It was found that PBOI-Cu (I) has a more compact structure and, consequently, a higher density than the membrane based on PAA-Cu (I). The sorption activity of the PAA-Cu (I) membrane is much higher in comparison with PBOI-Cu (I) due to the presence of functional groups in the structure of the PAA-Cu (I) monomer unit (Figure 3a). In pervaporation of methanol/MTBE mixture the PAA-Cu (I)-based membrane is more permeable and selective than the PBOI-Cu (I) membrane (Figure 3b). The separation factor and the total flux through the PAA-Cu (I) membrane have moderate values compared to the previously published data (Table 6).

**Figure 4:**
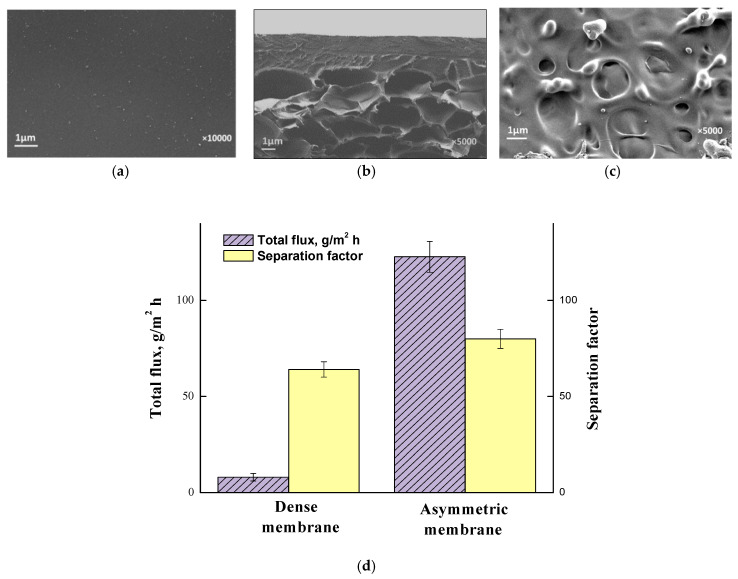
SEM images of asymmetric membrane: (**a**) top layer, (**b**) cross-section and (**c**) bottom. Total flux and the separation factor of asymmetric and dense membranes in the pervaporation of azeotropic methanol/MTBE mixture (14.3:85.7 wt.%) (**d**) [54].

For the first time, dense and asymmetric membranes based on the industrial polymer poly (4,4′-oxydiphenylene-pyromellitimide) (PMDA-ODA or Kapton) were studied for purifying MTBE from methanol by Pulyalina et al. [54]. When forming the asymmetric membrane, the authors were able to obtain an anisotropic structure consisting of a thin dense selective layer (3 μm) and a sponge-like microporous substrate (Figure 4a–c). The main transport parameters—the total flux and the separation factor of the asymmetric membrane—were higher than that of the dense membrane. Thus, the total flux for the asymmetric membrane is ~15 orders of magnitude greater than for the dense membrane (Figure 4d). Compared with published data, the PMDA-ODA asymmetric membrane has high performance and good separation efficiency.

In summary, the analysis of diffusion membranes based on polyheteroarylenes indicates the prospects for using these materials in the pervaporation separation of methanol/MTBE mixture. Formation of the membranes based on polymer-metal complexes (PAA-Cu (I)) leads to the highest separation efficiency in MTBE purification, while fabrication of a PA/PES-PEG composite membrane allows the highest separation performance to be obtained.

### 2.6. Others

In this section, some other polymeric materials and membranes that were also proposed for the separation of methanol/MTBE mixture will be considered. In the recent work [55], thin polymer films obtained by plasma polymerization of AA (acrylic acid) on a substrate of poly (3-hydroxybutyrate) (PHB) were obtained and characterized. The materials obtained in an inductively coupled radiofrequency plasma reactor retained certain functional groups of polyacrylic acid, but had a more complex chemical structure that varied depending on the operating conditions of the reactor. The designed membranes were selective towards methanol; the separation factor of modified materials over the entire concentration range was higher than for pure PHB membranes. The total flux, in turn, was lower than that of the pristine membrane; however, the modified membranes had better values of the pervaporation separation index (IPR). The permeability data for the modified membranes ranged from 660 to 4045 Barrer for methanol, while for MTBE they were significantly lower, namely from 10.5 to 57.7 Barrer. These large differences led to high membrane selectivity which ranged from 24 to 79.

In the earlier work [56] Villegas et al. performed the experiments on the sorption and pervaporation using membranes also based on PHB. The membrane selectivity for methanol was studied in the temperature range 25–50 °C. The polymer membrane with a 50% degree of crystallinity had relatively high-performance values for its application in the membrane separation technology of liquid organic mixtures. The PHB membrane showed excellent fluxes (9–15 kg/m^2^ h in the temperature range 25–50 °C) and moderate separation factors (3–4) for the feed containing 40 wt.% methanol.

Zhang et al. [57] synthesized copolymers with different ratios of AA-MMA (methyl methacrylate)-BA (butyl acrylate) to obtain pervaporation membranes. When studying the IR spectra of the films based on the copolymers, it was found that the membranes with the high BA content were highly hydrophobic. Membrane swelling in MTBE increased with increasing the BA content in the copolymers. At the same time, with an increase in the MMA content in the copolymers, the swelling degree in methanol decreased. The membrane based on the AA-MMA-BA copolymers with a ratio of 2:2:1 had a higher separation factor and a lower methanol flux compared to other membranes.

Polylactide (PLA) as a natural pristine polymer was used for the preparation of dense membranes in the work [58]. It was found that these membranes were selective towards methanol, especially for trace amounts of methanol in MTBE (the separation factor was more than 30 and IPR more than 15 kg/m^2^h for 1 wt.% methanol in the feed). The mechanical strength of the PLA membranes decreased after their immersion in methanol/MTBE mixture, but remained sufficient for pervaporation of the mixtures containing up to 20 wt.% methanol.

A study of the membranes based on polylactide was also carried out by Galliano et al. in [59]. The authors paid special attention to the influence of the solvent evaporation time (0.5–7 min) on the morphology and mechanical properties of the membranes obtained from a solution of ethyl lactate. The results of IR spectra, measurements of contact angles and experiments on the membrane swelling confirmed the chemical stability and the suitability of using the obtained films for pervaporation. It was shown that the membrane being dried for 7 min was selective for the separation of methanol from MTBE (α ≈ 75), primarily in comparison with other polylactide membranes obtained using more dangerous traditional solvents such as toluene, tetrahydrofuran and chloroform. These results deserve attention, given the fact that the membranes were obtained using a biopolymer and a “green” solvent.

Ray S., Ray S.K. [60] synthesized three different acrylamide copolymers with different contents of 2–hydroxyethyl methacrylate (PAMHEMA–1, –2, and –3), and prepared membranes based on crosslinked (gel-like) copolymers. These membranes predominantly sorbed methanol and were also more permeable towards the alcohol molecules during diffusion experiments. It was shown that the crosslinking degree increased with an increase in the HEMA content in the membrane, which led to a decrease in the total flux through the membrane. Among the three membranes, the PAMHEMA-3 one (composition of the copolymer AM:HEMA is 0.556:0.444) showed the highest selectivity and acceptable total flux (511.7 and 9.9 g/m^2^h for 0.53 wt.% methanol in the feed).

Membranes based on the mixture of agarose and hydroxyethyl cellulose (HEC) were studied in [61]. These membranes are also of interest as selectivity towards methanol was noted for them. It was shown that the addition of HEC to the agarose-based membrane material improved the membrane performance.

Asymmetric membranes based on poly(vinyl acetate) (PVAc) and PVP on an aluminum substrate were developed and characterized by Yoshida et al. in their study [62]. The active separation layer was formed by free-radical transplantation polymerization of PVAc and PVP on a vinylsilane-modified aluminum oxide substrate with an average pore diameter of 50 Å. The separation factors for PVP and PVAc-grafted pervaporation membranes reached values of 26 and 100, respectively, in the range of the methanol concentrations from 1 to 5 vol.%. The total fluxes through the PVAc and PVP-based membranes were from 0.055 to 1.26 and from 0.55 to 6.19 kg/m^2^h, respectively, for the methanol concentration range of 1–90 vol.%.

Separation of methanol/MTBE mixtures using the films of poly(ethylene-co-vinyl acetate) (EVAc) with different copolymer contents (9, 14, 19 and 28 wt.% of vinyl acetate) was investigated in [63]. In the presence of a liquid mixture, the membrane swelling degree increased as the vinyl acetate content in the membrane or MTBE concentration in the mixture increased. This behavior can be explained by two main factors: the polymer crystallinity degree and the difference in the solubility parameters of the liquids and the copolymer macromolecules. The pervaporation results indicate higher methanol permeability compared to MTBE, which is associated with higher methanol diffusion coefficients. At the same time, the selectivity of the process is also determined by a higher degree of interaction between the polymer and MTBE compared to methanol. As a result, low separation factors were obtained for all membranes at high productivity. The pervaporation results indicate higher methanol permeability compared to MTBE, which is associated with the higher methanol diffusion coefficients. At the same time, the membrane selectivity is also determined by a higher degree of interaction of the polymer with MTBE than with methanol. As a result, the low separation factors were obtained for all the membranes at high fluxes.

Doghieri et al. [37] studied the membranes based on poly(phenylene oxide) modified by introducing hydroxy groups into the main chain. Before pervaporation experiments, the membranes were subjected to thermal treatment for 1 h at 190 °C and kept in methanol/MTBE mixture (7 wt.%) for 10 days. In the course of the experiments, the membranes did not lose their mechanical strength and maintained stable transport properties. One of the factors affecting the separation process was plasticization occurred in the membrane with an increase in the methanol content in the feed, which led to an increase in the permeability of both components of the feed. For the asymmetric membranes with a selective layer thickness of 0.4 μm, the methanol flux was 4700 g/m^2^h when the feed contained 1.7 wt.% methanol. The separation factor in this case exceeded 20.

Separation of methanol/MTBE solutions was studied by Chen and Martin [64] using composite membranes obtained by depositing a thin selective layer of polystyrenesulfonate (PSS) on the surface of a microporous alumina substrate. To obtain a film, the synthesized poly(styrene-co-styrenesulfonic acids) were converted either to Na^+^ or to Mg^2+^-the counterionic form. It is shown that the composite membranes are highly selective towards methanol in comparison with MTBE. The methanol concentration in the permeate exceeded 99.5 wt.% for all the studied compositions of the feed. The authors note that the membranes in the ionic form of Mg^2+^ are characterized by higher separation factors than membranes containing Na^+^ as a counterion. The extremely high separation factors (from 25,000 to 35,000) were obtained for the PSS-Mg/Al_2_O_3_ composite membrane, but the total flux turned out to be rather low.

Zereshki et al. studied the properties of membranes made of modified poly(ether ether ketone) poly(oxa-p-phenylene-3,3-phthalido-p-phenylene-oxa-p-phenyleneoxy-phenylene) (PEEKWC) using chloroform as a solvent under varying separation conditions: temperature, flow rate and feed concentration [65]. The authors paid special attention to the high stability of the material during the pervaporation process. Despite the fact that the mechanical strength of the PEEKWC membranes slightly decreased upon the contact with a methanol/MTBE solution at high methanol concentrations, the obtained membranes had increased selectivity compared to commercial analogues. When studying the influence of the feed methanol content on the membrane performance, it was shown that the separation factor changed dramatically from 254 in the case of low methanol content to 6.6 at 22 wt.% alcohol, and then to 3.2 at 54 wt.% methanol. When separating the azeotropic mixture, the separation factor value was 14. The total flux gradually increased within the range of 0.015–0.113 kg/m^2^h with an increase in the feed methanol concentration.

Thus, composite membrane based on polystyrene sulfonate (PSS-Mg/Al_2_O_3_) demonstrates the extremely high separation factors, while dual-layer membrane PAA/PHB expectedly shows greater total flux (Table 7).

## 3. Mixed Matrix Membranes

One approach to the development of novel membranes is to use a polymer-inorganic nanocomposite. Membranes containing nanofillers (usually inorganic) that are dispersed in a polymeric material are classified as mixed matrix membranes (MMMs) [66]. Inorganic membranes generally have good mechanical stability and high performance, but their application is limited by their high cost and some undesirable performance characteristics, such as brittleness. When developing MMMs, certain problems occur, related to the compatibility between inorganic and organic components and the formation of defects in the material structure. Therefore, a significant part of the research on pervaporation of MMMs is aimed at studying and overcoming these problems [67]. As a result of the research, a number of studies related to the use of MMMs for the separation of methanol/MTBE mixture have been published. The typical inorganic nanofillers used for MMMs include zeolites, silicone, silicone dioxide, different metal oxides (ZrO_2,_ TiO_2_, Al_2_O_3_, ZnO), etc. Surface modification of nanofillers can also play an important role in improving membrane performance due to the possibility of improving affinity and compatibility of fillers with polymer matrix, changing the internal free volume, which, in turn, allows the permeability and selectivity to be controlled.

Khayet et al. estimated the effect of organosilane modified silica fillers on the pervaporation properties of dense membranes based on poly (2,6-dimethyl-1,4-phenylene oxide) (PPO) [68]. The authors investigated the crystallinity degree, thermal and mechanical properties of pure PPO and PPO modified by silicon dioxide nanoparticles. It was established that surface modification of the hydrophilic nanoparticles of inorganic silicon dioxide by organosilanes reduces the number of the surface silanol groups and leads to the formation of a stable silane network on the surface of silicon dioxide. It was proven by the measuring of membrane contact angles and SEM. The XRD spectra and the mechanical strength of the pristine and modified PPO were identical, while the thermal stability of the membrane modified with silicon dioxide nanoparticles was higher. According to the data on the sorption experiments using methanol/MTBE mixtures, the sorption degree increased with increasing the methanol concentration, and this parameter for methanol was higher in the case of the modified membrane. The authors explain this fact by the predominant influence of the diffusion stage in comparison with sorption of the molecules. The modified silicon dioxide nanoparticles have higher compatibility with the PPO polymer than unmodified ones, which provides the formation of more tortuous transport channels in the dense polymer matrix of PPO, reduces the diffusion rate of the components, and, consequently, their flow.

Membranes based on CA containing zeolite filler HZSM5 were studied by Ma et al. in [69]. The effect of the HZSM5 content on the sorption, diffusion, and pervaporation characteristics in the separation of methanol/MTBE mixture was evaluated. With an increase in the HZSM5 content in the membrane, the separation factor changed nonmonotonically, and the total flux increased. The methanol diffusion coefficients far exceeded this parameter for MTBE, which determined the high membrane selectivity. The composite containing 0.2 wt.% HZSM5 showed the highest separation factor of 346 at the total flux of 226 g/m^2^h (20 wt.% methanol, 30 °C).

Wang et al. [17] developed a novel CA membrane modified with the particles of metal oxides—Al_2_O_3_ and ZnO. The SEM and Raman spectrometry data showed that metal oxide particles were uniformly dispersed in the membrane matrix, contributing to an increase in both the total flux and the separation factor. Compared to the pure CA membrane, the maximum value of the total flux for the hybrid membrane filled with Al_2_O_3_ and ZnO increased to 2.5 and 3.5 kg/m^2^h, respectively; the maximum separation factor reached 859 and 772, respectively. In addition to the significantly improved separation efficiency, CA-based membranes with incorporated metal oxides proved to be inexpensive and have a relatively simple molding process.

In the study performed by Tamaddondar et al. [70], membranes with the strongly crosslinked nanosized selective layers were developed via layer-by-layer (LBL) assembly of polyanionic and cationic surfactants. Complex surfactant composite membranes (PELSCs) were prepared by depositing dilute and concentrated ionic solutions of sodium cellulose sulfate (NaCS) and hexadecylpyridinium chloride (HDPC) on PAN ultrafiltration membrane substrates. Moreover, PELSC nanocomposite membranes containing various amounts of the nanosized particles of SiO_2_ were first manufactured by injection molding. The study of the morphology confirmed the homogeneous structure of the membranes and the successful incorporation of nanosilica particles into the matrix. It was noted that an increase in the content of silicon nanodioxide from 2 to 10 wt.% in the composites led to an improvement in the permeability due to a decrease in the selectivity. This behavior was caused by an increase in the free volume, as well as possible defects at the interface between polymer and silicon clusters. The maximum total flux (1.62 kg/m^2^h) was obtained for the PELSC nanocomposite membrane containing 10 wt.% silicon dioxide nanoparticles.

Wang and co-authors [71] proposed a new approach for preparing hybrid membranes. GO nanoplates were modified in situ by polymerized hyperbranched poly(methylene bisacryl amide amino ethyl piperazine) (HPMA) via electrostatic interactions. The hyperbranched polymer provides the presence of terminal functional groups and the internal free volume in the polymer matrix, which allows controlling the permeability of small molecules. The obtained material was used to prepare membranes on ceramic tubular substrates using the vacuum assembly method. The methanol content in permeate and the total flux reached 99.5 wt.% and 0.41 kg/m2 h, respectively, with a methanol concentration in the feed of 10 wt.% at 40 °C. Hybrid membranes also showed a long operating time and good stability during 120-h testing.

Metal-organic frameworks (MOFs) were used as a new type of filler for hybrid membranes: MOFs were synthesized based on [Cu_2_(bdc)_2_(bpy)]_n_ with their subsequent introduction in SPES-C [72]. It was found that [Cu_2_(bdc)_2_(bpy)]_n_ predominantly sorbed methanol in comparison with MTBE. Both the sorption and diffusion selectivity increased with the incorporation of [Cu_2_(bdc)_2_(bpy)]_n_, which led to an increase in the separation factor. With an increase in the content of [Cu_2_(bdc)_2_(bpy)]_n_ in the polymer from 5 to 20 wt.%, the total flux increased to 0.288 kg/m^2^h at the separation factor of 1870.

The performance properties of various MMM membranes are presented in Table 8. It should be noted that the introduction of both inorganic and hybrid modifiers can significantly change the transport properties of the polymer matrix. Dual-layer membrane based on HPMA/GO demonstrates the highest performance among the described MMMs.

## 4. Conclusions

**Figure 5:**
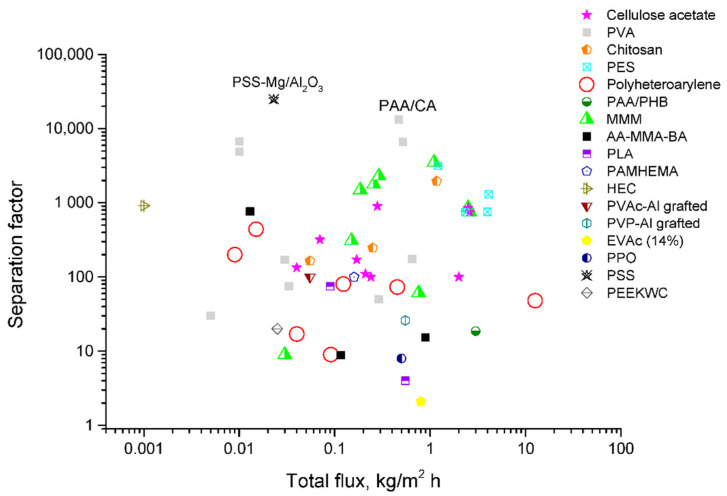
Comparison of the transport characteristics in the separation of methanol/MTBE mixture for the known membranes.

The analysis of the recent literature data allowed evaluating the prospects of using the pervaporation method for the separation of methanol/MTBE mixture in the MTBE producing technology. Most of the studies presented in the review, indeed, point to favorable forecasts. Figure 5 shows the ratio of the total flux and the separation factor for all the membranes considered in the article which were used or proposed for the separation of methanol/MTBE mixture. The presented data showed that a controlled change in the structure and transport properties of the membrane is possible using such modification methods as treatment by organic solvents, introduction of non-volatile additives, fabrication of polymer blends consisting of polymer mixtures, crosslinking and heat treatment. Applying inorganic and hybrid modifiers and creating mixed matrix membranes (MMMs) are also highly effective in the improvement of pervaporation performance. Today, the most promising materials with optimal transport properties for MTBE purification are not only PVA, but also modified cellulose acetate, polyheteroarylenes and mixed matrix membranes on their basis. In general, in the case of pervaporation separation, an inverse relationship between permeability and selectivity is usually observed: the higher the total flux, the worse the separation efficiency. The optimization of this ratio, namely the solution of the problem of increasing selectivity without a significant decrease in permeability (flux values), allows increasing the process efficiency in a view of modern technological problems. It should be noted that most of the membranes with high selectivity presented in the review are dense films, and the creation of composite membranes based on them will significantly increase the flux.

The most optimal transport properties are possessed by a composite membrane consisting of a selective layer based on crosslinked PVA supported on CA substrate. The extremely high separation factor was shown by a membrane based on polystyrene sulfonate (PSS-Mg/Al_2_O_3_).

## Figures and Tables

**Table 1:** Physical properties of methanol and methyl tert-butyl ether (MTBE) [3].

Liquid Mol. Weight, g/mol	Liquid Mol. Weight, g/mol	Molar Volume, cm^3^/mol	Density, g/cm^3^	T_boiling_, °C	Dynamic Viscosity, mPa∙s (20 °C)
Methanol	32.04	40.5	0.792	64.7	0.59
MTBE	88.15	119.0	0.740	55.2	0.35

**Table 2:** Transport properties of some membranes based on cellulose acetate used for the separation of methanol/MTBE mixture.

Membrane	T, °C	Methanol in the Feed, wt.%	Methanol in Permeate, wt.%	Total Flux, kg/m^2^h	Separation Factor	Ref.
CTA	40	10	92.6	0.21	110	[14]
CA-acetone (solvent)	-	6	89.5	0.04	134	[15]
CA-DMFA (solvent)	-	4	93.0	0.07	319	[15]
CA-NMP (solvent)	-	5	90	0.17	171	[15]
CA/PVP (85:15)	30	10	99.0	0.28	900	[16]
CA/CAHP	40	20	96.2	0.24	100	[20]
CTA (43.4% acet.)	40	20	96.2	2	100	[21]
CA316	50	20	94.8	0.47	75	[30]
CTA	40	22	95.1	2.27	69	[31]

**Table 3:** Transport properties of the membranes based on polyvinyl alcohol (PVA) used for the separation of methanol/MTBE mixture.

Membrane	T, °C	Methanol in the Feed, wt.%	Methanol in the Permeate, wt.%	Total Flux, kg/m^2^h	Separation Factor	Ref.
PAA/PVA (90:10)	35	5	89.9	0.03	170	[22]
PVA/PAA (80:20)	30	5	99.7	0.01	6700	[23]
PVA/SSA (95:5)	30	10	98.3	0.01	4900	[23]
PVA/PAN *	25	7	99.8	0.52	6630	[24]
PVA/CA *	25	7	99.9	0.47	13,272	[24]
PAA/PVA (COOH/OH = 0.26)	30	10	89.3	0.033	75	[32]
PVA/PAA (80:20)	25	5	61.2	0.005	30	[33]
PVA/CA (25%)	45	15	99.6	0.796	1427	[34]
PVA	25	10	95.3	0.65	175	[35]
PVAH-I	32	11	86.1	0.290	50	[36]

* Composite membrane.

**Table 4:** Transport properties of the membranes based on chitosan (CS) used for the separation of methanol/MTBE mixture.

Membrane	T, °C	Methanol in the Feed, wt.%	Methanol in the Permeate, wt.%	Total Flux, kg/m^2^h	Separation Factor	Ref.
PIC/PS *	40	25	98.8	0.25	247	[40]
28%PVP	30	14.45	96.5	0.055	164	[42]
CS/DSS (0.005 wt.%) *	25	20	95	0.8	76	[43]
SLS–chitosan/PES *	50	20	97.8	1.17	1956	[44]

* Composite membrane.

**Table 5:** Transport properties of the membranes based on polethersulfone (PES) used for the separation of methanol/MTBE mixture.

Membrane	T, °C	Methanol in the Feed, wt.%	Methanol in the Permeate, wt.%	Total Flux, kg/m^2^h	Separation Factor	Ref.
PES-C	40	15	99.3	2.35	747	[45]
PES-C/PVP (24 wt.%)	40	15	99.2	3.98	754	[45]
PES-C	40	5	99.4	1.21	3124	[46]
SPES-C (SD = 0.64)	40	15	99.6	4.1	1300	[47]

**Table 6:** Transport properties of the membranes based on polyheteroarylenes used for the separation of methanol/MTBE mixture.

Membrane	T, °C	Methanol in the Feed, wt.%	Methanol in the Permeate, wt.%	Total Flux, kg/m^2^h	Separation Factor	Ref.
PA-6-H	30	50	97.8	12.6	48	[49]
Matrimid	25	14.3	73.9	0.04	17	[50]
GO-polyimide	45	14.3	60	0.091	9	[51]
PA/PES-PEG	30	20	94.8	0.453	73	[52]
PAA-Cu(I)	50	10	98.0	0.015	440	[53]
PBOI-Cu(I)	50	10	95.8	0.009	200	[53]
Asymmetric PI-PM	50	14.3	89.9	0.123	80	[54]

**Table 7:** Transport properties of various polymer membranes used for the separation of methanol/MTBE mixture.

Membrane	T, °C	Methanol in the Feed, wt.%	Methanol in the Permeate, wt.%	Total Flux, kg/m^2^h	Separation Factor	Ref.
PPO (OH-modified)	22	20	67	0.5	8	[37]
PAA/PHB *	40	20	82.3	3.0	18.6	[55]
AA–MMA–BA (2:2:1)	30	3	96	0.013	764	[57]
AA–MMA–BA (1:1:1)	30	3	32	0.897	15.3	[57]
AA–MMA–BA (1:1:2)	30	3	21	0.116	8.8	[57]
PLA	30	10	30.9	0.55	4	[58]
PLA/ethyl lactate	35	14.3	92.6	0.09	75	[59]
PAMHEMA	30	10	91.8	0.16	100	[60]
HEC/Aga (1:1)	30	9	98.9	0.001	912	[61]
PVAc-Al grafted	20	1	50.3	0.055	100	[62]
PVP-Al grafted	20	5	57.8	0.55	26	[62]
EVAc (14%)	20	10	19	0.8	2.1	[63]
PSS-Mg/Al_2_O_3_ *	25	14.4	99.9	0.023	25,000	[64]
PEEKWC	30	10	68.9	0.025	20	[65]

* Composite membrane.

**Table 8:** Transport properties of some hybrid membranes used for the separation of methanol/MTBE mixture.

Membrane	T, °C	Methanol in the Feed, wt.%	Methanol in the Permeate, wt.%	Total Flux, kg/m^2^h	Separation Factor	Ref.
CA-Al_2_O_3_ (2%)	40	31	99.7	2.5	859	[17]
CA-ZnO (4%)	40	31	99.7	2.6	750	[17]
PPO-Si	25	10	50	0.03	9	[68]
CA-HZSM5 (0.2%)	30	10	97.3	0.15	310	[69]
PELSC/PAN *	25	14.3	91.1	0.76	61	[70]
HPMA/GO *	40	10	99.7	1.1	3500	[71]
SPES-C/[Cu_2_(bdc)_2_(bpy)]_n_ (5%)	40	15	99.6	0.185	1490	[72]
SPES-C/[Cu_2_(bdc)_2_(bpy)]_n_ (15%)	40	15	99.7	0.257	1780	[72]
SPES-C/[Cu_2_(bdc)_2_(bpy)]_n_ (30%)	40	15	99.75	0.29	2300	[72]

* Composite membrane.

## References

[1] Seyferth D. (2003). The Rise and Fall of Tetraethyllead. 2. Organometallics.

[2] Thornton S.F., Nicholls H.C.G., Rolfe S.A., Mallinson H.E.H., Spence M.J. (2020). Biodegradation and fate of ethyl tert-butyl ether (ETBE) in soil and groundwater: A review. J. Hazard. Mater..

[3] Lide D.R., Haynes W.M.M., Baysinger G., Berger L.I., Frenkel M., Goldberg R.N., Kehiaian H.V., Kuchitsu K., Roth D.L., Zwillinger D. (2010). CRC Handbook of Chemistry and Physics.

[4] Sundmacher K., Kienle A. (2006). Reactive Distillation: Status and Future Directions.

[5] Hauan S., Hertzberg T., Lien K.M. (1997). Multiplicity in reactive distillation of MTBE. Comput. Chem. Eng..

[6] Krupinova O.N., Zhuchkov V.I., Frolkova A.K. (2015). Synthesis and discrimination of process flow sheets for the separation of a reaction mixture of methyl tert-butyl ether production. Theor. Found. Chem. Eng..

[7] Abu Al-Rub F.A., Abdel-Jabbar N., Darwish N., Ghanem H. (2002). Vapor-Liquid Equilibrium of MTBE-Methanol, MTBE-Methanol-Calcium Chloride and MTBE-Methanol-Lithium Chloride Mixtures. Chem. Eng. Technol..

[8] Coto B., Mössner F., Pando C., Rubio R.G., Renuncio J.A.R. (1997). Vapor-liquid equilibrium of the methanol [1,1-dimethylethyl methyl ether (MTBE) or 1,1-dimethylpropyl methy ether (TAME)] systems. Fluid Phase Equilib..

[9] Gmehling J., Menke J., Krafczyk J., Fischer K. (1995). A data bank for azeotropic data—Status and applications. Fluid Phase Equilib..

[10] Górak A., Sørensen E. (2014). Distillation: Fundamentals and Principles.

[11] Hamid H., Ali M.A. (2004). Handbook of MTBE and Other Gasoline Oxygenates.

[12] Bitar L.S., Hazbun E.A., Piel W.J. (1984). MTBE production and economics. Hydrocarb. Process..

[13] Noureddini H. (2001). Ethyl tert-Butyl Ether and Methyl tert-Butyl Ether: Status, Review, and Alternative Use Exploring the Environmental Issues of Mobile, Recalcitrant Compounds in Gasoline. ACS Symp. Ser..

[14] Yang J.S., Kim H.J., Jo W.H., Kang Y.S. (1998). Analysis of pervaporation of methanol-MTBE mixtures through cellulose acetate and cellulose triacetate membranes. Polymer.

[15] Tabe-Mohammadi A., Villaluenga J.P.G., Kim H.J., Chan T., Rauw V. (2001). Effects of polymer solvents on the performance of cellulose acetate membranes in methanol/methyl tertiary butyl ether separation. J. Appl. Polym. Sci..

[16] Wu H., Fang X., Zhang X., Jiang Z., Li B., Ma X. (2008). Cellulose acetate-poly(N-vinyl-2-pyrrolidone) blend membrane for pervaporation separation of methanol/MTBE mixtures. Sep. Purif. Technol..

[17] Wang Y., Yang L., Luo G., Dai Y. (2009). Preparation of Cellulose Acetate Membrane Filled with Metal Oxide Particles for the Pervaporation Separation of Methanol/Methyl Tert-Butyl ether Mixtures. Chem. Eng. J..

[18] Cao S., Shi Y., Chen G. (1999). Pervaporation separation of meOH/MTBE through CTA membrane. J. Appl. Polym. Sci..

[19] Zhang L., Chen H.-L., Zhou Z.-J., Lu Y., Gao C.-J. (2002). Pervaporation of methanol/MTBE/C5 ternary mixtures through the CA membrane. Desalination.

[20] Niang M., Luo G., Schaetzel P. (1997). Pervaporation separation of methyl tert-butyl ether/methanol mixtures using a high-performance blended membrane. J. Appl. Polym. Sci..

[21] Cao S., Shi Y., Chen G. (2000). Permeation behaviour in cellulose triacetate dense membrane during pervaporation separation of methanol/methyltert-butyl ether mixture. Polym. Int..

[22] Park H.C., Ramaker N.E., Mulder M.H.V., Smolders C.A. (1995). Separation of MTBE—Methanol Mixtures by Pervaporation. Sep. Sci. Technol..

[23] Rhim J.W., Kim Y.K. (2000). Pervaporation separation of MTBE-methanol mixtures using cross-linked PVA membranes. J. Appl. Polym. Sci..

[24] Bangxiao C., Li Y., Hailin Y., Congjie G. (2001). Effect of separating layer in pervaporation composite membrane for MTBE/MeOH separation. J. Memb. Sci..

[25] Kim S.G., Kim Y.-I., Yun H.G., Lim G.T., Lee K.H. (2003). Preparation of asymmetric PVA membranes using ternary system composed of polymer and cosolvent. J. Appl. Polym. Sci..

[26] Lux S., Winkler T., Forstinger M., Friesenbichler S., Siebenhofer M. (2015). Pervaporative Separation of Methanol–Methyl Acetate Mixtures with Commercial PVA Membranes. Sep. Sci. Technol..

[27] Gaálová J., Vojtek L., Lasnier S., Tadic T., Sýkora J., Izák P. (2019). Separation of Trimethyl Borate from a Liquid Mixture by Pervaporation. Chem. Eng. Technol..

[28] Li W., Luis P. (2018). Understanding coupling effects in pervaporation of multi-component mixtures. Sep. Purif. Technol..

[29] Edgar K.J., Buchanan C.M., Debenham J.S., Rundquist P.A., Seiler B.D., Shelton M.C., Tindall D. (2001). Advances in cellulose ester performance and application. Prog. Polym. Sci..

[30] Cao S., Shi Y., Chen G. (2000). Influence of acetylation degree of cellulose acetate on pervaporation properties for MeOH/MTBE mixture. J. Memb. Sci..

[31] Niang M., Luo G. (2001). A triacetate cellulose membrane for the separation of methyl tert-butyl ether/methanol mixtures by pervaporation. Sep. Purif. Technol..

[32] Gozzelino G. (2005). Pervaporation of methanol/methyl-t-butyl ether mixtures through poly(vinyl alcohol)/poly(acrylic acid) blend membranes. Sep. Sci. Technol..

[33] Hilmioglu N.D., Tulbentci S. (2004). Pervaporation of MTBE/methanol mixtures through PVA membranes. Desalination.

[34] Zhou K., Zhang Q.G., Han G.L., Zhu A.M., Liu Q.L. (2013). Pervaporation of water-ethanol and methanol-MTBE mixtures using poly (vinyl alcohol)/cellulose acetate blended membranes. J. Memb. Sci..

[35] Peivasti M., Madandar A., Mohammadi T. (2008). Effect of operating conditions on pervaporation of methanol/methyl tert-butyl ether mixtures. Chem. Eng. Process. Process. Intensif..

[36] Singha N.R., Kar S., Ray S.K. (2009). Synthesis of novel polymeric membrane for separation of MTBE-methanol by pervaporation. Sep. Sci. Technol..

[37] Doghieri F., Nardella A., Sarti G.C., Valentini C. (1994). Pervaporation of methanol-MTBE mixtures through modified poly(phenylene oxide) membranes. J. Memb. Sci..

[38] Kumar M.N.V.R., Muzzarelli R.A.A., Muzzarelli C., Sashiwa H., Domb A.J. (2004). Chitosan chemistry and pharmaceutical perspectives. Chem. Rev..

[39] Han Y.J., Wang K.H., Lai J.Y., Liu Y.L. (2014). Hydrophilic chitosan-modified polybenzoimidazole membranes for pervaporation dehydration of isopropanol aqueous solutions. J. Memb. Sci..

[40] Kim S.G., Kim Y.-I., Lim G.T., Jegal J., Lee K.H. (2002). Polyion complex composite membranes for the separation of methyl t-butyl ether/methanol mixtures: Separation behaviors of these membranes. J. Appl. Polym. Sci..

[41] Kim S.G., Kim Y.-I., Jegal J., Lim G.T., Lee K.H. (2002). Characterization and preparation of polyion complex composite membranes for the separation of methyl tert-butyl ether/methanol mixtures. J. Appl. Polym. Sci..

[42] Cao S., Shi Y., Chen G. (1999). Properties and pervaporation characteristics of chitosan-poly(N-vinyl-2-pyrrolidone) blend membranes for MeOH-MTBE. J. Appl. Polym. Sci..

[43] Huang R.Y.M., Moon G.Y., Pal R. (2001). Chitosan/anionic surfactant complex membranes for the pervaporation separation of methanol/MTBE and characterization of the polymer/surfactant system. J. Memb. Sci..

[44] Yong Nam S., Moo Lee Y. (1999). Pervaporation separation of methanol/methyl t-butyl ether through chitosan composite membrane modified with surfactants. J. Memb. Sci..

[45] Blanco J.F., Nguyen Q.T., Schaetzel P. (2001). Novel hydrophilic membrane materials: Sulfonated polyethersulfone Cardo. J. Memb. Sci..

[46] Han G.L., Zhang Q.G., Zhu A.M., Liu Q.L. (2013). Pervaporation separation of methanol/methyl tert-butyl ether mixtures using polyarylethersulfone with cardo membranes. Sep. Purif. Technol..

[47] Han G.L., Zhang Q.G., Liu Q.L. (2013). Separation of methanol/methyl tert-butyl ether using sulfonated polyarylethersulfone with cardo (SPES-C) membranes. J. Memb. Sci..

[48] Pulyalina A.Y., Polotskaya G.A., Toikka A.M. (2016). Membrane materials based on polyheteroarylenes and their application for pervaporation. Russ. Chem. Rev..

[49] Kopeć R., Meller M., Kujawski W., Kujawa J. (2013). Polyamide-6 based pervaporation membranes for organic-organic separation. Sep. Purif. Technol..

[50] Castro-Muñoz R., Galiano F., Fíla V., Drioli E., Figoli A. (2018). Matrimid^®^5218 dense membrane for the separation of azeotropic MeOH-MTBE mixtures by pervaporation. Sep. Purif. Technol..

[51] Castro-Muñoz R., Galiano F., de la Iglesia Ó., Fíla V., Téllez C., Coronas J., Figoli A. (2019). Graphene oxide—Filled polyimide membranes in pervaporative separation of azeotropic methanol–MTBE mixtures. Sep. Purif. Technol..

[52] Alibakhshian F., Pourafshari Chenar M., Asghari M. (2019). Thin film composite membranes with desirable support layer for MeOH/MTBE pervaporation. J. Appl. Polym. Sci..

[53] Pulyalina A., Polotskaya G., Goikhman M., Podeshvo I., Gulii N., Shugurov S., Tataurov M., Toikka A. (2017). Preparation and characterization of methanol selective membranes based on polyheteroarylene—Cu(I) complexes for purification of methyl tertiary butyl ether. Polym. Int..

[54] Pulyalina A., Tataurov M., Faykov I., Rostovtseva V., Polotskaya G. (2020). Polyimide Asymmetric Membrane vs. Dense Film for Purification of MTBE Oxygenate by Pervaporation. Symmetry.

[55] Villegas M., Romero A.I., Parentis M.L., Castro Vidaurre E.F., Gottifredi J.C. (2016). Acrylic acid plasma polymerized poly(3-hydroxybutyrate) membranes for methanol/MTBE separation by pervaporation. Chem. Eng. Res. Des..

[56] Villegas M., Vidaurre E.F.C., Habert A.C., Gottifredi J.C. (2011). Sorption and pervaporation with poly(3-hydroxybutyrate) membranes: Methanol/methyl tert-butyl ether mixtures. J. Memb. Sci..

[57] Zhang L., Chen H.L., Pan Z.R. (2003). Study on swelling behavior and pervaporation properties of AA-MMA-BA copolymers for separation of methanol/MTBE/C5 mixtures. J. Appl. Polym. Sci..

[58] Zereshki S., Figoli A., Madaeni S.S., Simone S., Drioli E. (2010). Pervaporation separation of methanol/methyl tert-butyl ether with poly(lactic acid) membranes. J. Appl. Polym. Sci..

[59] Galiano F., Ghanim A.H., Rashid K.T., Marino T., Simone S., Alsalhy Q.F., Figoli A. (2019). Preparation and characterization of green polylactic acid (PLA) membranes for organic/organic separation by pervaporation. Clean Technol. Environ. Policy.

[60] Ray S., Ray S.K. (2006). Synthesis of highly methanol selective membranes for separation of methyl tertiary butyl ether (MTBE)–methanol mixtures by pervaporation. J. Memb. Sci..

[61] Yoshikawa M., Yoshioka T., Fujime J., Murakami A. (2002). Pervaporation of methanol/methyl tert-butyl ether mixtures through agarose/hydroxyethylcellulose blended membranes. J. Appl. Polym. Sci..

[62] Yoshida W., Cohen Y. (2003). Ceramic-supported polymer membranes for pervaporation of binary organic/organic mixtures. J. Memb. Sci..

[63] Gozzelino G., Malucelli G. (2004). Permeation of methanol/methyl-t-butyl ether mixtures through poly(ethylene-co-vinyl acetate) films. Colloids Surf. A Physicochem. Eng. Asp..

[64] Chen W.J., Martin C.R. (1995). Highly methanol-selective membranes for the pervaporation separation of methyl t-butyl ether/methanol mixtures. J. Memb. Sci..

[65] Zereshki S., Figoli A., Madaeni S.S., Simone S., Esmailinezhad M., Drioli E. (2011). Pervaporation separation of MeOH/MTBE mixtures with modified PEEK membrane: Effect of operating conditions. J. Memb. Sci..

[66] Chung T.S., Jiang L.Y., Li Y., Kulprathipanja S. (2007). Mixed matrix membranes (MMMs) comprising organic polymers with dispersed inorganic fillers for gas separation. Prog. Polym. Sci..

[67] Vane L.M. (2018). Review: Membrane materials for the removal of water from industrial solvents by pervaporation and vapor permeation. J. Chem. Technol. Biotechnol..

[68] Khayet M., Villaluenga J.P.G., Valentin J.L., López-Manchado M.A., Mengual J.I., Seoane B. (2005). Filled poly(2,6-dimethyl-1,4-phenylene oxide) dense membranes by silica and silane modified silica nanoparticles: Characterization and application in pervaporation. Polymer.

[69] Ma X., Hu C., Guo R., Fang X., Wu H., Jiang Z. (2008). HZSM5-filled cellulose acetate membranes for pervaporation separation of methanol/MTBE mixtures. Sep. Purif. Technol..

[70] Tamaddondar M., Pahlavanzadeh H., Saeid Hosseini S., Ruan G., Tan N.R. (2014). Self-assembled polyelectrolyte surfactant nanocomposite membranes for pervaporation separation of MeOH/MTBE. J. Memb. Sci..

[71] Wang L., Wang N., Yang H., An Q., Li B., Ji S. (2018). Facile fabrication of mixed matrix membranes from simultaneously polymerized hyperbranched polymer/modified graphene oxide for MTBE/MeOH separation. J. Memb. Sci..

[72] Han G.L., Zhou K., Lai A.N., Zhang Q.G., Zhu A.M., Liu Q.L. (2014). [Cu2(bdc)2(bpy)]n/SPES-C mixed matrix membranes for separation of methanol/methyl tert-butyl ether mixtures. J. Memb. Sci..

